# Rats with steroid-induced polycystic ovaries develop hypertension and increased sympathetic nervous system activity

**DOI:** 10.1186/1477-7827-3-44

**Published:** 2005-09-07

**Authors:** Elisabet Stener-Victorin, Karolina Ploj, Britt-Mari Larsson, Agneta Holmäng

**Affiliations:** 1 Cardiovascular Institute and Wallenberg Laboratory, Sahlgrenska Academy, Göteborg University, SE-413 45 Göteborg, Sweden; 2 Department of Obstetrics and Gynaecology, Sahlgrenska University Hospital, Sahlgrenska, SE-413 45 Göteborg, Sweden; 3 Institute of Occupational Therapy and Physical Therapy, Sahlgrenska Academy, Göteborg University, SE-405 30 Göteborg, Sweden

## Abstract

**Background:**

Polycystic ovary syndrome (PCOS) is a complex endocrine and metabolic disorder associated with ovulatory dysfunction, abdominal obesity, hyperandrogenism, hypertension, and insulin resistance.

**Methods:**

Our objectives in this study were (1) to estimate sympathetic-adrenal medullary (SAM) activity by measuring mean systolic blood pressure (MSAP) in rats with estradiol valerate (EV)-induced PCO; (2) to estimate alpha_1a _and alpha_2a _adrenoceptor expression in a brain area thought to mediate central effects on MSAP regulation and in the adrenal medulla; (3) to assess hypothalamic-pituitary-adrenal (HPA) axis regulation by measuring adrenocorticotropic hormone (ACTH) and corticosterone (CORT) levels in response to novel-environment stress; and (4) to measure abdominal obesity, sex steroids, and insulin sensitivity.

**Results:**

The PCO rats had significantly higher MSAP than controls, higher levels of alpha_1a _adrenoceptor mRNA in the hypothalamic paraventricular nucleus (PVN), and lower levels of alpha_2a _adrenoceptor mRNA in the PVN and adrenal medulla. After exposure to stress, PCO rats had higher ACTH and CORT levels. Plasma testosterone concentrations were lower in PCO rats, and no differences in insulin sensitivity or in the weight of intraabdominal fat depots were found.

**Conclusion:**

Thus, rats with EV-induced PCO develop hypertension and increased sympathetic and HPA-axis activity without reduced insulin sensitivity, obesity, or hyperandrogenism. These findings may have implications for mechanisms underlying hypertension in PCOS.

## Introduction

Polycystic ovary syndrome (PCOS), a heterogeneous endocrine and metabolic disorder affecting 6% to 10% of women of reproductive age, is associated with ovulatory dysfunction, abdominal obesity, hyperandrogenism, and in many cases hypertension and profound insulin resistance [1]. Several of these factors increase the risk of cardiovascular disease (CVD) in women [2]. The prevalence of hypertension in women with PCOS is around 40% [3,4]. PCOS is also associated with a higher risk of myocardial infarction (relative risk, 7.4) [4] and, in young women, a compromised cardiovascular profile, independent of obesity [5]. Women with PCOS also develop a hyperactive response to stress [6]. The heterogeneity of the disorder suggests that there are subpopulations within the syndrome. Hypertension and insulin resistance are not uniformly present, and hypertension may be absent despite profound insulin resistance and vice versa [7]. It is not clear whether PCOS increases the risk of CVD independently of the metabolic syndrome [8].

The abnormalities detected in PCOS have been attributed to primary defects in the hypothalamic-pituitary-adrenal (HPA) axis, the ovarian microenvironment, the adrenal gland, and the insulin/insulin-like growth factor metabolic regulatory system [1]. Hypertension and defects in insulin action may be related to enhanced sympathetic-adrenal medullary (SAM) activity [9]. Increased sympathetic nervous system activity may be associated with enhanced α-adrenergic responsiveness, contributing to elevated blood pressure and hypertension [10,11], and may play a role in PCOS [6,12,13]. If so, the α_1a _and α_2a _adrenoceptor (AR) subtypes are of interest since they are strongly implicated in cardiovascular control [14,15].

The etiology of PCOS is unknown, likely reflecting multiple pathophysiological mechanisms, and an accepted animal model of the disease has not been established. A recent review concluded that multiple models may be needed, depending on whether the goal is to investigate ovarian morphology or a particular PCOS-related disorder [16]. In both mechanistic and treatment studies, we and others have used a model in which PCO is induced by a single intramuscular (i.m.) injection of estradiol valerate (EV) in 8-week-old rats [17]. The rats cease ovulating and develop characteristics of human PCOS, including large cystic follicles in the ovaries and altered concentrations of luteinizing hormone [17].

Central neuronal activity in norepinephrine (NE) neurons is increased in rats with EV-induced PCO [18], suggesting increased central sympathetic outflow. Increased ovarian sympathetic tone in rats with EV-induced PCO has been evidenced by elevations in tyrosine hydroxylase activity and NE concentration, downregulation of the β_2 _AR, and increased production of ovarian nerve growth factor, a target-derived neurotrophin [19-25].

The stress response is largely mediated by the SAM and HPA axes. Activation of the HPA axis increases the secretion of adrenocorticotropic hormone (ACTH) and leads to glucocorticoid release from the adrenal cortex. The HPA axis and intra-adrenal mechanisms involving the adrenal medulla might also regulate adrenocortical steroidgenesis. Furthermore, adrenocortical steroids influence the development and the function of adrenomedullary chromaffin cells and vice versa [26]. Activation of the SAM axis, however, increases the release of epinephrine and NE from the adrenal medulla and stimulates the sympathetic norepinergic nerves, increasing NE secretion.

These two axes form the key efferent links in the "defeat" (HPA axis) and "defense" (SAM axis) reactions [27,28]. Chronic activation of either axis can lead to stress-related diseases such as hypertension, diabetes, and obesity [27,28].

In this study, we investigated SAM-axis activity and HPA-axis regulation in rats with EV-induced PCO. To estimate SAM activity, we measured systolic blood pressure and the expression of α_1a_- and α_2a_-AR mRNAs in the hypothalamic paraventricular nucleus (PVN) and adrenal medulla. To assess HPA-axis regulation, we measured ACTH and corticosterone (CORT) concentrations in response to novel-environment stress. We also measured abdominal obesity, sex steroid levels, and insulin sensitivity.

## Materials and methods

### Animals

Eighteen virgin adult cycling Wistar-Kyoto rats (B & K Universal AB, Sweden) weighing 190 to 210 g were divided into two groups and housed four to a cage under standard conditions (21 ± 2°C, 50% to 60% humidity, 12-hour light/12-hour dark cycle) for at least 1 week before and throughout the study, with free access to standard chow and tap water. The study was approved by the Animal Ethics Committee of Göteborg University and performed in accordance with the NIH "Guide for the Care and Use of Laboratory Animals."

### Hormonal Treatment and Study Procedure

After 1 week of acclimatization, 8-week-old rats (n = 10) each received an i.m. injection of EV (Riedeldehaen, Germany), 4 mg in 0.2 mL of oil (arachidis oleum, Apoteket AB, Umeå, Sweden), to induce PCO [17]; this dose induces persistent estrus and permanent PCO, i.e. no fresh corpora lutea, regressing old corpora lutea and atretic follicles [22]. Controls (n = 8) received vehicle only. Rats were weighed weekly. Mean systolic arterial pressure (MSAP) and heart rate (HR) were measured 2, 3, 5, and 7 weeks after the injection. Stress tests were carried out at 6 weeks. Blood samples for measurement of progesterone, 17β-estradiol, and testosterone were obtained at week 7. Euglycemic hyperinsulinemic clamp tests were performed at 10 to 11 weeks, the time point when PCO morphology is fully developed[22].

### Vaginal Smear

For 10 days before the stress and clamp tests, estrous cyclicity was monitored by vaginal smears obtained between 0800 and 1200 hours. The rat estrous cycle (estrus, diestrus 1, diestrus 2, and proestrus) usually lasts about 4 days. In controls, the stress and clamp tests were performed during estrus, and sex hormone levels were measured during estrus. In PCO rats, all test procedures were carried out and blood samples collected during estrus or pseudoestrus as described [19]

### Blood Pressure and HR Measurements

Systolic arterial pressure and HR were measured between 0800 and 1200 hours at 2, 3, 5, and 7 weeks after EV injection with a tail cuff monitor (RTBP Monitor, Harvard Apparatus, South Natick, MA) with a light-emitting diode and a photoresistor connected to a dual-channel recorder. The rats were conscious and placed on a heating pad, and their tails were warmed with a heating lamp for 10 minutes to cause vasodilatation for an optimal signal. MSAP was calculated from three consecutive stable recordings.

### ACTH and CORT Secretion

At 6 weeks, ACTH and CORT responses to novel-environment stress were assessed as described [29]. All tests started between 0700 and 0900 h, and care was taken to keep the rats undisturbed and fed the night before the experiment. The next morning, each rat was placed in a novel environment (new test cage, loud background noise). Blood (60 μL) for measurement of ACTH and CORT was obtained from a tail vein before and 15, 30, 60, 90, and 120 minutes after exposure to the novel environment.

### Euglycemic Hyperinsulinemic Clamp Test

At 10 to 11 weeks after injection, rats underwent euglycemic hyperinsulinemic clamp tests as described [30]. Anesthesia was induced with thiobutabarbital sodium (Inactin, 125 mg/kg body weight; RBI, Natick, MA), and catheters were inserted into the left carotid artery for blood sampling and into the right jugular vein for glucose and insulin infusions. Rats were placed on a heating pad to maintain a constant rectal temperature of 37.0 ± 0.1°C.

After a bolus injection (Actrapid, 100 U/mL, Novo, Copenhagen, Denmark), insulin was infused at 8 mU/kg per minute. A 20% glucose solution in physiological saline was administered to maintain plasma glucose at 7 mM; the infusion rate was guided by glucose measurements in 10-μL blood samples obtained every 5 minutes until a steady state was achieved (~60 minutes) and then every 10 minutes. The mean infusion rate was calculated from values during the last 60 minute. At 0 and 120 minutes, 250-μl blood samples were obtained to measure insulin concentration. Less than 1 mL of blood was taken from each rat.

### Tissues

After the clamp test, rats were decapitated, the hypothalamus was quickly removed and dissected on dry ice at the border of -0.8–-3.8 according to rat brain stereotactic coordinates [31]. Tissue punches centered on PVN were taken from the sections using an 18 gauge needle, and the adrenal medullae were dissected and snap frozen in liquid nitrogen until assay. The parametrial, mesenteric, retroperitoneal, and inguinal adipose tissues, one ovary and muscles of the hind limb (extensor digitorum longus, soleus, and tibialis anterior) were rapidly dissected by the same person and weighed.

### Ovarian morphology

The ovary was removed, cleaned of adherent connective fat tissue, and fixed in 4% formaldehyde buffer for at least 24 hours. Thereafter the samples were dehydrated and imbedded in paraffin. The ovaries were partially longitudinally sectioned (4 μm, every tenth section mounted on the glass slide) and stained with hematoxylin and eosin. An experienced pathologist, blinded to grouping analysed the follicle population under microscope. There was no intention to quantify the number of growing or atretic follicles but rather to establish whether ovulation with corpora lutea formation had occurred within the given time frame. According to morphometric (stereological) and statistical principles there is no need to perform a statistical analysis in this situation.

### Real-Time PCR Analysis

Total RNA from the PVN and adrenal medulla was extracted with RNeasy Mini kits (Qiagen, Hilden, Germany). First-strand cDNA was synthesized from 1 μg of total RNA with TaqMan reverse transcription reagents (Applied Biosystems., Foster City, CA). Each 100 μl RT-PCR reaction contained 1 μg of total, 1 × TaqMan RT buffer, 5 mM MgCl2, 2.5 mM random hexamers, 1 mM dNTP, 0.4 U/ml RNase inhibitor, and 1.25 U/ml Multiscribe RT (PE Applied Biosystems, Foster City, CA, USA). Reverse transcription was carried out in a PTC-200 PCR system (MJ Research., Boston, MA, USA) at 25°C for 10 min, 48°C for 30 min and 95°C for 5 min. PCR was performed with the ABI Prism 7700 sequence detection system and FAM-labeled probes specific for the α_1a _AR (NM 017191) and α_2a _AR (rCT57545) (PE Applied Biosystems, Stockholm, Sweden). Designed primers and a VIC-labeled probe for GAPDH (NM_031144) were included as internal standards. cDNA was amplified for one cycle of 50°C for 2 minutes and 95°C for 10 minutes, followed by 40 cycles of 95°C for 15 seconds and 60°C for 1 minute. mRNA levels were calculated with the standard curve method (User Bulletin 2, PE Applied Biosystems) and adjusted for GAPDH expression.

### RIA and ELISA

Blood was collected into potassium EDTA tubes (ACTH), heparinized microtubes (CORT and insulin), or ordinary tubes (steroids) and centrifuged immediately at 4°C in a microcentrifuge. RIA kits were used to measure ACTH (DSL-2300, Diagnostic Systems Laboratories, Webster, TX), CORT (RSL^125^I Corticosterone RIA kit; MP Biomedicals, Costa Mesa, CA), testosterone (DSL-4100, Diagnostic Systems Laboratories), progesterone (DSL-3400, Diagnostic Systems Laboratories), and estradiol-17β (double-antibody estradiol procedure, DPC Scandinavia AB, Mölndal, Sweden). Insulin levels were determined by ELISA (Merkodia, Uppsala, Sweden).

### Statistical Analysis

All statistical analyses were performed with SPSS 11.0 software. Body weight and hemodynamic data were analyzed by repeated-measures ANOVA. Weight gain, tissue weight, glucose infusion rate and glucose and insulin levels, stress and sex hormones, and mRNA levels were analyzed by two-tailed *t *test. Values are expressed as mean ± SE. *P *< 0.05 was considered significant.

## Results

### PCO Rats Develop Hypertension

**Figure 1:**
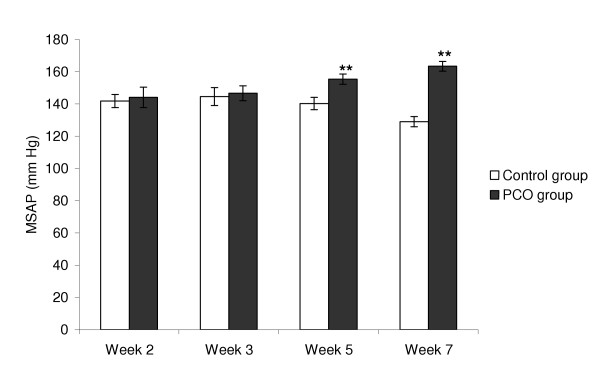
MSAP 2, 3, 5 and 7 weeks after EV injection. At both time points, MSAP was significantly higher in PCO rats than controls. Values are mean ± SE. **P < 0.01 versus controls (*t *test).

At 5 and 7 weeks, MSAP was significantly higher in the PCO group than in controls (Figure 1). At 7 weeks, HR was also significantly higher in the PCO group (405 ± 13.6 versus 345 ± 10.0 beats/minute, *P *< 0.001).

### Expression of AR mRNA in the Hypothalamic Paraventricular Nucleus and Adrenal Medulla

**Figure 2:**
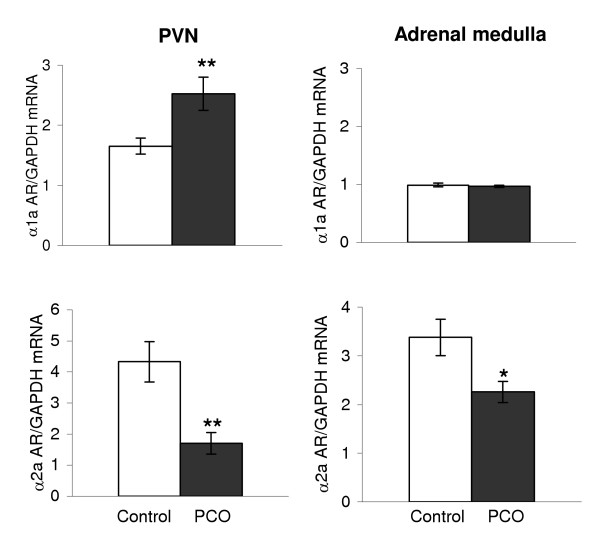
Expression of α_1a _and α_2a _AR mRNA in the hypothalamic paraventricular nucleus (PVN) and adrenal medulla; values are normalized to GAPDH expression. Expression of α1_a _AR mRNA was upregulated in the PVN. Expression of α_2a _AR mRNA was downregulated in both the PVN and adrenal medulla. Values are mean ± SE. *P < 0.05, **P < 0.01 versus controls (*t *test.

Expression of α_1a _AR mRNA in the PVN was significantly higher, and expression of α_2a _AR mRNA in the PVN and adrenal medulla significantly lower, in the PCO group than in controls (Figure 2).

### ACTH and CORT Responses to Stress Are Higher in PCO Rats

To assess effects of EV exposure on the HPA axis, we measured the ACTH and CORT responses to novel-environment stress at 6 weeks (Table 2). At baseline, ACTH levels were similar in the two groups, while CORT levels tended higher in PCO rats. At 15 and 30 minutes, ACTH levels were higher in PCO rats than controls, but the difference was significant (*P *< 0.05) only when the two time points were analyzed together (not shown). At 30 and 60 minutes, CORT levels were significantly higher in the PCO group.

**Table 2:** Plasma ACTH and CORT Concentrations before and in Response to Novel Environment Stress Test 6 Weeks after EV Injection

Hormone	Control group	PCO group	*P*
ACTH (pmol/L)			
0 min	56.48 ± 5.97	52.21 ± 1.99	n.s.
15 min	79.83 ± 3.97	93.59 ± 10.71	n.s.
30 min	76.57 ± 7.04	97.45 ± 12.48	n.s.
60 min	77.85 ± 10.26	76.47 ± 7.84	n.s.
90 min	70.68 ± 3.31	72.23 ± 6.33	n.s.
120 min	68.34 ± 4.09	75.84 ± 4.38	n.s.
			
CORT (ng/mL)			
0 min	251.32 ± 46.05	416.44 ± 93.29	n.s.
15 min	597.9 ± 41.26	756.44 ± 95.47	n.s
30 min	668.63 ± 46.18	1001.85 ± 132.73	0.05*
60 min	657.09 ± 65.02	980.83 ± 151.58	0.05*
90 min	744.51 ± 82.48	754.73 ± 129.68	n.s
120 min	537.63 ± 44.23	873.56 ± 158.80	n.s.

Values are mean ± SE. n.s. = non significant.

*t test.

### PCO Rats Have Lower Testosterone and Higher Progesterone Levels

The PCO group had significantly lower testosterone and higher progesterone concentrations than the controls (Table 3). There were no differences in 17β-estradiol concentrations.

**Table 3:** Estradiol, Testosterone, and Progesterone Concentrations 7 Weeks after EV Injection

Hormone	Control group	PCO group	*P*
17β-estradiol (pmol/L)	0.23 ± 0.02	0.22 ± 0.03	n.s.
Testosterone (nmol/L)	0.45 ± 0.04	0.27 ± 0.02	0.01
Progesterone (nmol/L)	12.78 ± 1.10	22.54 ± 3.38	0.05

Values are mean ± SE. n.s. = not significant.

### PCO Rats Have Normal Insulin Sensitivity

The glucose infusion rate and plasma insulin and glucose concentrations before and during the clamp test are shown in Table 4. Insulin sensitivity was not significantly different in the PCO and control groups, as reflected by the glucose infusion rate.

**Table 4:** Glucose Infusion Rate and Plasma Insulin and Glucose Concentrations before and at Steady State during the Euglycemic Hyperinsulinemic Clamp Test

Measurement	Oil group	PCO group	*P*
Glucose infusion rate (mg/kg/min)*	26.6 ± 1.0	26.3 ± 1.1	n.s.
Glucose level (mmol/L)			
0 min	6.5 ± 0.2	6.3 ± 0.2	n.s.
120 min	6.8 ± 0.1	6.7 ± 0.1	n.s.
Insulin level (mU/mL)			
0 min	85 ± 17	69 ± 10	n.s.
120 min	280 ± 38	274 ± 66	n.s.

Values are mean ± SE. n.s. = not significant (*t *test).

*At steady state (60–120 min).

### Ovarian morphology – day 60

**Figure 3:**
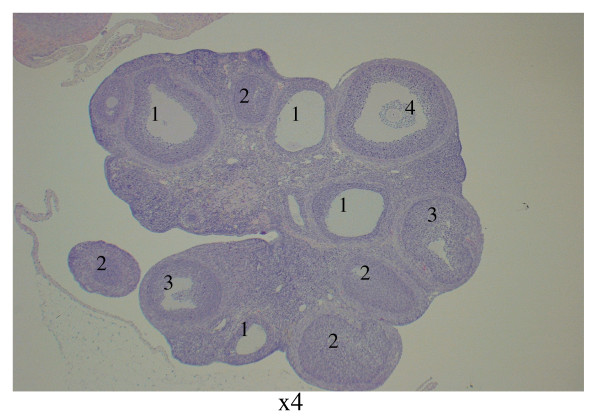
PCO group control group 60 days after EV injection. Survey view showing atretic follicles (1), regressing old corpora lutea (2), growing "healthy" follicles (3), and atretic secondary follicle with detachment of the oocyte from the cumulus mass of pycnotic granulosa cells (4) ×4 obj.

The ovaries in the control group exhibited a typically normal appearance with follicles and corpora lutea in different stages of development and regression. The ovaries in the PCO group displayed typical PCO-like changes [17], and both the number and size of the corpora lutea in these groups were decreased compared with the control group. Typically, no young corpora lutea were present in the PCO groups. The dominant structures were atretic follicles, regressing old corpora lutea, and a few growing "healthy" follicles in the primary, secondary, and tertiary stages (Figures 3).

### PCO Rats Gain Less Total Body Weight

From weeks 3 to 10, PCO rats weighed less than controls, but the difference was significant only between weeks 3 and 6 (Table 1). Overall, the PCO group gained significantly less weight than the controls.

**Table 1:** Total Body Weight during the Study Period

	Body weight (g)		
		
Week	Control group	PCO group	*P*
2	188.9 ± 1.6	192.1 ± 0.9	n.s.
3	205.7 ± 1.7	195.8 ± 1.1	0.001*
4	212.6 ± 1.6	203.6 ± 2.6	0.01*
5	214.9 ± 1.8	208.1 ± 2.6	0.05*
6	221.3 ± 2.3	213.0 ± 2.5	0.05*
7	220.8 ± 2.5	215.5 ± 2.8	n.s.
8	221.3 ± 2.3	216.4 ± 2.5	n.s.
9	225.0 ± 2.5	218.6 ± 2.6	n.s.
10	232.9 ± 3.3	228.5 ± 2.8	n.s.
Weight gain (g/week)	4.7 ± 0.2	3.9 ± 0.3	0.05^†^

Values are mean ± SE. n.s. = not significant.

*Repeated-measures ANOVA.

^†^*t *test.

### No Differences in Intraabdominal Fat Depots or Hindlimb Skeletal Muscles

The inguinal fat depot (representing subcutaneous fat), was significantly heavier in the PCO group than in the controls (4.36 ± 0.2 and 3.36 ± 0.2 g/kg body weight, *P *< 0.01). No differences were observed in the weights of the mesenteric, parametrial, and retroperitoneal fat depots (representing intraabdominal fat) or the hind limb skeletal muscles (not shown).

## Discussion

This study shows that rats with EV-induced PCO developed hypertension, consistent with increased sympathetic activity, as in human PCOS. They also had increased expression of α_1a _AR mRNA in PVN and decreased expression of α_2a _AR mRNA in PVN and adrenal medulla, consistent with elevated blood pressure and increased sympathetic activity, together with hyperactivity of the HPA and SAM axes. Rats with EV-induced PCO did not have reduced insulin sensitivity or become obese or hyperandrogenic.

### Sympathetic Activity and the SAM Axis

Women with PCOS have a high incidence of hypertension [3,4] and are at increased risk of CVD [32]. The sympathetic nervous system might play a crucial role in the development of hypertension and CVD. As an example, hypertension is common in patients with polycystic kidney disease (PKD) and a recent study by Klein *et al*. demonstrates that their muscle sympathetic nerve activity is increased regardless of renal function [11]. This support the hypothesis that sympathetic hyperactivity also may contribute to the pathogenesis of hypertension in PCOS. In addition, overactivity of the SAM axis increases blood pressure [9]. Since sympathetic activity and hypertension are strongly correlated, we measured MSAP and found that it was increased at 5 and 7 weeks after EV injection. This finding may have implications for the mechanisms underlying the increased risk of developing hypertension, including the higher incidence of CVD later in life, in women with PCOS.

In the PCO group, α_1a _AR mRNA expression was increased in the PVN, and α_2a _AR expression was reduced both in the PVN and adrenal medulla, strongly suggesting increased sympathetic activity. In spontaneously hypertensive rats, increased central α_1a _AR expression and decreased α_2a _AR expression correlated with elevated blood pressure and increased sympathetic activity [15]. In another study, Peng *et al*. showed that the α_2a _AR has a sympathoinhibitory role in the brain [33]. Furthermore, α_2 _AR subtypes have been demonstrated to control the release of catecholamines from sympathetic nerves and from adrenal medulla [34]. Since the hypothalamus participates in central regulation of MSAP and adrenal medulla is involved in the regulation of SAM-axis, these findings support the conclusion that EV-induced PCO is associated with increased sympathetic activity.

The PCO rats gained significantly less weight than controls. The findings of enhanced adrenal glucocorticoid production following EV-injection in the present study might explain the lack of weight gain in PCO rats via lipolytic actions. Activation of the α_1 _and α_2 _ARs in the hypothalamic paraventricular nucleus inhibits food intake [35], suggesting that the dysregulated central expression of α_1 _and α_2 _ARs in the PCO group was responsible for the difference in weight gain. Food intake was not measured. It is important to keep in mind that PCOS in women is not always associated with obesity [36]. However, increased sympathetic nervous activity has been associated with increased metabolic activity, fat consumption, and decreased body weight, but not with reduced food intake [37].

In a study of cardiovascular risk factors, young women with PCOS had a higher prevalence of hypertension than population controls [5] and a compromised cardiovascular risk profile, even after adjustment for their higher BMI. In a recent study, low birth size and final height predicted high sympathetic nerve activity in adulthood [38] and this was positively correlated to high MSAP. Interestingly, low birth weight has been pointed out as an increased risk of developing PCOS [39]. Thus, the increased CVD risk associated with PCOS cannot be explained by obesity alone. These findings suggest that the greater risk reflects increased sympathetic nerve activity, which in turn results in hypertension.

Signs of hyperactivation in EV-induced PCO include downregulation of β_2 _AR in theca-interstitial cells [40]. Reduced expression of the β_2 _AR also enhances progesterone secretion by cystic ovaries in response to isoproterenol in rats with EV-induced PCO [19]. In the current study, the PCO group had significantly higher progesterone concentrations than the controls. In addition, high levels of P and low levels of T suggest a metabolic shift towards corticosterone synthesis and secretion and a hyper-adrenal state.

All these changes seemed to be independent of 17β-estradiol, since they occurred without any changes in its concentrations [41]. Consistent with this finding, 17β-estradiol concentrations were unaltered in the PCO group.

### Interactions Between the SAM and HPA Axes

The novel-environment stress test also provided evidence for increased activity of the HPA axis in EV-induced PCO. The PCO group had higher CORT levels at 30 and 60 minutes than controls. Corticotropin-releasing homone (CRH) and locus ceruleus – NE (sympathetic nervous system) have been pointed out as important components of the stress system as well as their peripheral effectors, the HPA and SAM axes [42]. The CRH and locus ceruleus – NE systems directly modulate the HPA and SAM axes in order to maintain homeostasis [42]. Rats with EV-induced PCO have higher CRH levels in the median eminence than controls [43]. In the present study, the development of hypertension, increased expression of α_1a _AR and decreased expression of α_2a_-AR mRNA, and increased CORT responses to stress indicate a close interaction between the HPA and SAM axes, as well as increases in their activity. The interesting finding that the CORT response to stress was higher whereas the ACTH response was lower then expected can be explained by direct stimulation of adrenal glucocorticoids with less pituitary ACTH action [44].

### Insulin Resistance, Hyperandrogenism, and Obesity

According to one theory of the pathogenesis of PCOS, a defect in insulin action leads to hyperinsulinemia and insulin resistance [45]. The insulin clamp tests performed 10 to 11 weeks after EV injection, when typical PCO are fully developed [17], showed no insulin resistance in the PCO group.

Why didn't our rats with EV-induced PCO develop insulin resistance? One possibility is that the measurements was done prior to the onset of insulin resistance. Another possibility is absence of intraabdominal obesity, thought to be a contributing factor [1]. The major abnormality in insulin action in PCOS is believed to be a post-receptor defect in the insulin signaling cascade in skeletal muscle, which might be due to interaction with testosterone [1]. In humans, fasting insulin levels correlate positively with androgen levels, and some studies have shown that hyperandrogenism causes insulin resistance in humans [46] and in rats [30]. Plasma testosterone levels were low in the PCO group, consistent with the lack of insulin resistance or hyperinsulinemia. As mentioned before, low levels of T suggest a metabolic shift towards corticosterone synthesis and secretion and a hyper-adrenal state.

It is also important to remember that only about 50% of women with PCOS have insulin resistance and no more than about 40% are obese [47]. Moreover, there are pitfalls in assessing insulin resistance in PCOS, including the lack of consensus on what defines PCOS and "normal" insulin sensitivity, ethnic and genetic variability, confounding factors such as obesity, stress, and aging, and concerns about whether simplified models of insulin sensitivity have the precision to predict treatment needs, responses, and morbidity [48].

### Perspectives

Different models of PCO are inevitable, and the model to use depends on the goals of the study. We demonstrated that rats with EV-induced PCO develop hypertension, with increased α_1a _AR and decreased α_2a _AR mRNA expression, consistent with increased sympathetic activity. We also found evidence of increased HPA-axis activity.

These findings may have implications for mechanisms underlying hypertension in women with PCOS, since essential hypertension is associated with sympathetic hyperactivity, which is itself known to increase CVD risk. We also demonstrated that rats with EV-induced PCO do not have reduced insulin sensitivity and do not develop obesity or hyperandrogenism, which might be later signs of sympathetic hyperactivity. Hyperglycemia of diabetic PCOS patients has been found to be significantly positively correlated with adrenal hypersecretion of cortisol, dehydroepiandrostenedione (DHEA) and dehydroepiandrostenedione sulfate (DHEAS) [49]. They suggested that enhanced adrenocortical (HPA) activity may be important factor underlying the development of type 2 diabetes in women with PCOS. Since we found exaggerated activity in both HPA and SAM axes in the present EV-induced PCO model, it might mimic an initial stage of PCOS.

These findings might provide important leads for future studies with the EV-induced PCO model.

## Authors' contributions

ES-V participated in the design of the study, carried out part of the animal preparation, performed RT-PCR, performed the statistical analysis and drafted the manuscript. KP participated in the design of the study and carried out the animal preparation. B-M carried out the animal preparation. AH participated in the design of the study and in writing the manuscript. All authors read and approved the final manuscript.
